# Living life with cerebral palsy? A description of the social safety nets for individuals with cerebral palsy in the Nordic countries

**DOI:** 10.1177/1403494820974564

**Published:** 2020-12-15

**Authors:** Ann I. Alriksson-Schmidt, Ira Jeglinsky, Gudny Jonsdottir, Abdu Kedir Seid, Gunvor Klevberg, Eva Buschmann, Reidun Jahnsen

**Affiliations:** 1 Department of Clinical Sciences Lund, Orthopedics, Lund University, Skane University Hospital, Lund, Sweden; 2 Department of Health and Welfare, Arcada University of Applied Sciences, Helsinki, Finland; 3 Endurhaefing, Rehabilitation Centre of Excellence, Kopavogur, Iceland; 4 Centre for Alcohol and Drug Research, Aarhus University, Aarhus, Denmark; 5 Cerebral Palsy Follow-up Program, Department of Neurosciences for Children, Oslo University Hospital, Oslo, Norway; 6 The Norwegian Cerebral Palsy Association, Oslo, Norway; 7 Faculty of Medicine, Research Center of Habilitation and Rehabilitation Models and Services (CHARM), University of Oslo, Oslo, Norway

**Keywords:** Cerebral palsy, social benefits, environmental factors, disability, CPNorth

## Abstract

**Aims:**

This report reviews major laws, acts and regulations of social benefits and services for individuals with disabilities, focusing on cerebral palsy in the five Nordic countries. It summarizes the available benefits and services and the re-application process and provides comparative analyses among the countries.

**Methods:**

Published reports, articles and relevant government and municipal websites were reviewed for each respective country and used to compile an overview and comparison between the countries.

**Results:**

In the Nordic countries, there are a number of laws and regulations in place to support individuals with cerebral palsy and their families. In addition, there are numerous social benefits available for which individuals with disabilities can apply. Although there are national differences, the similarities across the five countries regarding laws, social benefits offered for individuals with cerebral palsy and the application processes are clear. However, the application processes seem cumbersome and, at times, redundant. Physicians and other healthcare specialists repeatedly need to write ‘medical certificates’ describing the diagnosis and its consequences for a disability that is chronic and lifelong.

**Conclusions:**

Participation in society for individuals with cerebral palsy disabilities can be enabled by social benefits. By extension, social benefits may indirectly have implications for public health in individuals with disabilities. Although the lives of individuals with cerebral palsy – as with others – can improve in certain areas, the need for social benefits will generally increase, not decrease, over time. Although it is clearly important to have checks and balances that prevent system misuse, it might be worthwhile from a cost-benefit perspective to investigate whether the current systems could be improved to better manage time and resources and avoid emotional distress by streamlining the application process.

## Background

The birth of a child with a disability changes the realities and perspectives of families. Clearly, to have a child with a disability does not have to be negative. At times, children with disabilities can bring unique positive effects and make special contributions to families and family life [1,2]. Having a child with a disability can, however, be time consuming, create emotional stress and contribute to financial vulnerability [3–6]. Caregivers will have to learn what types of health and social services are needed and available, how to apply for them, if and how their children’s pre-school or academic careers need to be modified, and how the home environment needs to be altered to fit the needs of the family. The extended care responsibilities do not diminish over time but are lifelong.

According to the ecological model, individuals are highly influenced by the different systems that surround them, which are referred to as micro-, meso- and macrosystems [7]. Environmental factors play a vital role in disability, as stressed in the World Health Organization’s (WHO’s) International Classification of Functioning, Disability and Health (ICF) [8]. The United Nations (UN) has defined disability as ‘the result of the interaction between persons with impairments and the environmental barriers that hinder their full and effective participation in society on an equal basis with others’ [9]. Environmental factors, such as social and public health policies, the interpretation and enforcement of laws, the availability of support systems, societal attitudes and the degree of universal design, greatly affect the lives of those in need of support due to their disability.

**Figure 1.**
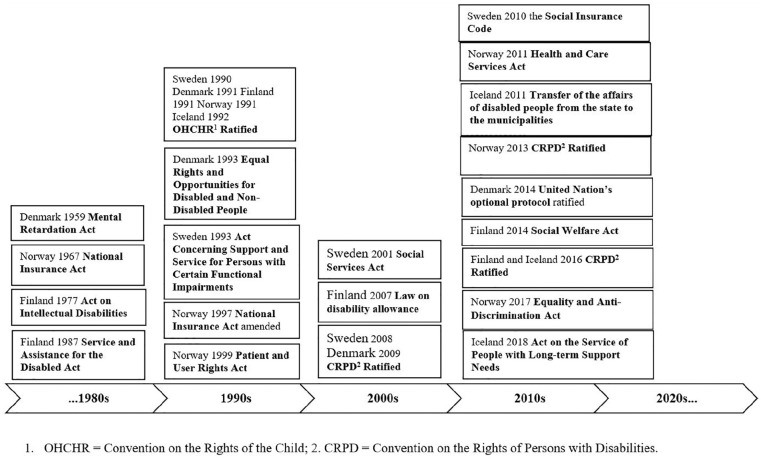
Examples of the major laws, acts and regulations related to social benefits in the context of cerebral palsy in the Nordic countries.

According to the WHO’s and the World Bank’s World Report on Disability, individuals with disabilities are some of the most marginalized people in society with limited access to education, employment and financial resources [9]. They are also more likely to be impoverished due to systematic barriers, stigma and alienation [9]. In the 1970 and 1980s, a shift from institutionalization to deinstitutionalization began to take hold in the industrialized world [10], with mainstreaming becoming a key principle. Although most institutions housed individuals with psychiatric or mental illnesses, individuals with physical/intellectual disabilities (IDs) were also admitted. The notion of equal rights for people with disabilities required the removal of hurdles, including legislative barriers. In 2006, the UN published the Convention on the Rights of Persons with Disabilities (CRPD), mandating at-home, residential and community support services to prevent isolation and segregation by supporting inclusion in the community and the promotion and protection of human rights and fundamental freedoms of all persons with disabilities [11]. All Nordic countries have ratified the CRPD. In 1989, the UN’s Convention on the Rights of the Child (OHCHR) was published and has been ratified by all the Nordic countries (Figure 1).

### Cerebral palsy and the Nordic context

One of the most common lifelong disabilities is cerebral palsy (CP). CP is caused by non-progressive brain damage that occurs before the age of two years [12]. There is great variability in functioning, but motor impairments are always present and challenges in cognition, perception, sensation, behaviour and comorbidities such as epilepsy are common [12,13]. For clinical and research purposes, gross motor function is classified using the Gross Motor Function Classification System (GMFCS; ages 0–12 years) and more recently the extended and revised version (ages 0–18 years) [14]. Distinctions between the five mutually exclusive GMFCS levels are based on everyday functional performance and use of assistive technology and the quality of movement [15]. GMFCS level I implies gross motor function is the least affected. The GMFCS levels have been reported to be stable, and there is less change for those at the ends of the classification system [16]. There are similar classification systems for hand function, the Manual Ability Classification System (MACS; ages 4–18 years) and the Mini-MACS for children younger than four years of age [17,18], and for communication the Communication Function Classification System (CFCS) [19], which, when combined, mirror the complexity of the condition. Secondary conditions that are preventable [20], such as musculoskeletal problems and pain, are frequent. The severity of CP in combination with comorbidities and secondary conditions, and how these interact with environmental factors, determine if persons with CP might need or be eligible for social benefits.

The environmental context in which individuals reside is central and in the Nordic countries, universal healthcare is the norm. There are similarities across the healthcare systems with multidisciplinary collaborations free of charge, regular follow-ups and the availability of free or greatly subsidized assistive devices, medications and treatments. Nevertheless, in a review of disability benefits for adults residing in seven European countries, including the Nordic countries, the authors reported great national differences in laws and regulations and the handling of social benefit claims [21]. The authors observed many of the identified problems were shared and emphasized that international exchange of experiences concerning benefits for people with disabilities was needed.

CPNorth – Living Life with Cerebral Palsy in the Nordic Countries? is a multinational research collaboration with more than 20 researchers and users (https://www.arcada.fi/en/research/project/cp-north) [22]. One of the objectives is to delineate social benefits available for individuals with CP in the Nordic countries and the overall purpose of the present report was to describe and compare the social benefits available for individuals with CP and their families in the Nordic countries. Social benefits are intertwined with public health in that access to social benefits facilitates access, inclusion and participation for individuals with CP. The goal was not to provide an exhaustive systematic review, but to compose a summary of central themes of the social benefits that are available, and how these social benefits/services are organized. The aims were to (a) outline major laws, acts and regulations related to social benefits for individuals with CP, (b) summarize what social benefits are available, what their central themes are and what agencies are responsible for these, (c) describe the process of applying for social benefits, including the renewal process and (d) provide comparative analyses among the countries. It should be noted that for consistency and given the scope of the report, we tend to refer to individuals with CP. Naturally, laws and regulations do not only apply to individuals with CP, they are also applicable for individuals with other types of disabilities. Thus, the terms individuals with CP and individuals with disabilities are used throughout the report.

## Methods

Social benefits were defined as financial support, services or assistance reimbursed by the government or municipalities, which may be applicable to individuals with CP. Healthcare and education services were excluded. Information was retrieved by (a) PubMed searches using the following search terms: disability, CP, social benefits, disability benefits; (b) accessing information on government and municipal websites; and (c) reviewing relevant published reports. An author fluent in the language of the specific country performed the search, then summarized and translated the findings. A user representative provided feedback on the manuscript. Because not all children live with their biological parents, the term caregiver is used in this article. In most cases, this is a biological, adoptive or foster parent. As this article has a lifespan approach, the term caregiver is used in relation to individuals with CP and their families and includes children and adults.

## Results

### Major laws, acts and regulations related to social benefits in the context of CP

All five countries have ratified the OHCHR and the CRPD (Figure 1).

#### Sweden

According to Swedish disability policy, disability should be considered broadly, not only with respect to health and medical care [23]. Individuals with CP are affected by numerous legislations, for instance the 2009 Discrimination Act. However, focus is placed on the complementary act created in 1993, the Act Concerning Support and Service for Persons with Certain Functional Impairments (LSS). The LSS applies to individuals with considerable or permanent functional impairments to ensure rights to basic measures when support provided by other laws is insufficient. The act does not consider the financial means of the families, but the needs of the person. It comprises 10 areas, of which four focus on the caregivers. Examples of services are advice and personal support, personal assistance or companion services, short ‘stays away’ from home, short periods of supervision for schoolchildren aged over 12 years and daily activities [24]. The costs associated with LSS are continually debated [25,26] and the number of applicants granted benefits under LSS has declined, or fewer services have been granted in recent years [27]. Changes to LSS and other legislations relevant to disability are underway.

#### Iceland

Social benefits and services for people with CP are primarily accommodated by local authorities, in line with the notion that services should be decentralized and provided locally [28]. Issues concerning people with disabilities fall under the Ministry of Social Affairs, although the Ministry of Health is responsible for central supervision of healthcare services. The primary legislation, the Act on the Service of People with Long-term Support Needs, outlines service provisions for people with CP. Its objective is for people with disabilities, including CP, to have access to the best services available, enjoy full human rights and equality with fellow citizens and facilitate independent living, including participation in policy making.

#### Finland

Finnish disability policy emphasizes that all of society should promote the rights of people with disabilities, ensure availability and high quality of services and increase accessibility in society [29]. Several laws influence the services for people with CP, including the Constitution of Finland and the Act of Equality between People. The Law on Disability Allowance aims to ensure sufficient allowance to support the ability to handle daily life, such as participating in education or the workforce, living at home and rehabilitation. Three main laws pertain to people with CP. The Service and Assistance for the Disabled Act promotes the conditions for people with CP and other disabilities to live as equal members of society. In the new proposal, individual needs will guide service planning. It also contains clarifications on personal assistance, housing, travel support and mobility. The Social Welfare Act defines how to enhance and maintain welfare and social security, reduce inequality and increase participation. This law is being revised by the Parliament. Finally, the Act on Intellectual Disabilities focuses on special needs, for example, supporting the ability to manage everyday life for people with IDs.

#### Denmark

The 1959 Mental Retardation Act provided those with IDs civil rights in almost all aspects. The Mental Retardation Service was then established under the jurisdiction of the Ministry of Social Affairs [30,31]. In 1993, an equal opportunity resolution was adopted for both public and private companies and institutions to apply the principle of equal rights and opportunities to people with disabilities. Denmark has acceded to the Additional Optional Protocol of the CRPD, allowing people with disabilities to file complaints regarding possible violations of the CRPD to the UN’s Disability Committee. According to the Ministry of Children and Social Affairs, the disability policy is based on the CRPD and four principles: the Principle of Equality, which implies equal treatment and opportunities; the Principle of Compensation, which means people with disabilities should be compensated to overcome barriers that prevent them from participating in community life; the Sector Responsibility Principle, which concerns the division of responsibility – all public authorities are responsible for making their services accessible to people with disabilities and to incorporate disability into policy development; finally, the Principle of Solidarity means most social services are paid through taxes and made available free of charge to the citizens who need them.

#### Norway

The most comprehensive welfare legislation for persons with disabilities, the National Insurance Act, was adopted in 1967 and amended in 1997. The law includes income protection (disability benefit) and compensates for expenses resulting from disability. The act is managed by the Labor and Welfare Administration, which is a state directorate with offices in all municipalities. Non-healthcare-related services include home care and help with housekeeping and personal assistance. According to the Health and Care Service Act from 2011, municipalities must offer respite to caregivers and be able to provide housing for those who need it. People with extensive care needs are entitled to coordinators and an Individual Service Plan (ISP). The precursor to the Health and Care Service Act was the Health Service Act in the municipalities (Municipal Health Service Law). The goal was to create more comprehensive health and social services, with municipalities responsible for planning and coordination. The financial responsibility was divided between states and municipalities. The health service providers in the municipalities consider the assistance needs an individual has. The number of hours approved is then decided, which can be appealed to the county governor. In 2015, user-controlled personal assistance (UPA) was adopted to strengthen participation and self-determination. Assistance with personal care and training, help at home and participation in activities, companion services and respite care is available through UPA. Finally, the Equality and Anti-Discrimination Ombud Act (disability, gender, sexual orientation and ethnicity), adopted in 2017, combines four previous laws into one. The act contains provisions on universal design and the right to individual adaptation in kindergarten, education and working life.

## Social benefits for people with disabilities, their families and the authorities responsible

A summary description of services and benefits by country is presented in Table I.

**Table I.** Names and descriptions of service or benefit by country.

Name of service/benefit^a^	Description of service/benefit	Sweden	Iceland	Finland	Denmark	Norway
Disability pension	Monthly retirement or disability pension.	Disability pension available. Depends on work ability and age. Generally applicable between 19–64 years.	Disability pension, age-related (aged 18–67 years) disability supplement and guaranteed income.	Disability pension for those aged >16 years. Depends on work ability. Based on care needs, not income.	Disability pension if you are over 40 and if work ability does not improve by treatment or other interventions.	Disability pension from 18–67 years if work ability is ⩽50%. Upper limit 66% of salary in past 5 years.
Respite care home	Respite care provided in private homes, in the home of the client, or in sheltered housing.	The service is available as “short stay away from home” for all 5 countries.				
User controlled personal assistant	Assistant working for an individual user (or family), where the user serves as the manager, deciding what services are provided.	User-controlled personal assistance available through private businesses or municipalities for all five countries.				
Support contact	A person to accompany and assist users to enable meaningful leisure-time and social contacts.	The support is available, primarily through LSS.	The support is available through the municipality.	The support is available through the municipality for ⩽30 hours per week.	The support is available through the municipality.	The support is available through the municipality.
Caregiver benefit	Compensation for loss of income when caring for a relative in need of full-time care and attention.	Temporary parental benefits are being replaced with care allowance for children with special needs and additional cost allowance with a disability (under revision).	Tax-free care allowance for caregivers of children <18 years. After age 18 years, there is disability pension.	The benefit is available through the municipality.	The benefit is available through the municipality.	The benefit is available through the municipality.
Attendance allowance	Entitled families with a child in need of extra care and supervision due to illness, injury, or disability.	Childcare allowance for care, supervision, and/or for added costs; temporary parental benefits.	Users can apply for partial refund for accommodation expenses.	Childcare allowance for children ⩽ 16.	Caregivers are allowed to train their child at home and will be compensated for lost income.	The allowance is available through NAV. No age limit if the child lives with the caregivers.
Basic benefits for all five countries	Cover necessary additional expenses incurred due to the child’s disability.	Available through childcare allowance for added costs related to special food, medicine, clothing and leisure activities.				
Training allowance (compensation for loss income)	Compensate for loss of income if a caregiver has to attend a course or training necessary to care for a child with disability.	Available through LSS (10 contact days per caregivers of children >16 years of age)	Parental payments for those who are unable to work or attend education due to their child with disability.	Special care allowance to compensate earning loss of caregivers.	Available, the amount depending on caregivers’ income.	Available on request for each course or training from NAV. No age limit if the child lives with the caregivers.
Transport and car subsidies	Financial grants to buy or modify a vehicle for persons who cannot use public transport.	Car allowance for those with severe mobility disability and for individuals older than 65 years. Renewed every 9 years (with exceptions based on annual mileage).	Refund for certain travel expenses. Car allowance to buy or modify a car, which is renewed every 5 years.	Free public transport and the municipality covers half of the purchase costs of a car.	Discount in public transport. Interest-free loan to buy a car and repaid in 8 years.	Reduced price on public transport. Car subsidies are available from NAV.
Housing grant	Grant aiming to improve accessibility for disabled persons.	Available for additional housing expenses. Dependent on income.	Refunds for accommodation costs for caregivers.	Individual housing and services support. From 2020, all individuals with disability will live in their homes instead of in institutions.	Available support and increased if a person or someone who lives with them gets help 24 hours a day.	Available for additional housing expenses, if low income, through the municipality.

a All benefits need to be applied for by the person with disability or their legal guardians and the social benefits may or may not be approved.

LSS: Act Concerning Support and Service for Persons with Certain Functional Impairments; NAV: Norwegian Labour and Welfare Administration.

### Sweden

The government, municipalities and county councils share the responsibilities of maintaining financial and social stability for individuals with CP [21]. If eligible for LSS, an ISP should be created with user input. The municipalities pay for the initial 20 hours of weekly assistance. If the Social Insurance Agency decides that more than 20 hours assistance per week are needed, the government pays the additional cost. The municipalities are responsible for most of the 10 areas in LSS, with the National Board of Health and Welfare providing central supervision. Services are generally free (and tax free), but municipalities may charge for certain things, such as meals when staying away from home.

Caregivers of children with certain disabilities can apply for benefits in addition to LSS. If a child or adolescent ⩽19 years of age needs long-term care or supervision, caregivers may apply for a childcare allowance for care and supervision from the Social Insurance Agency. If there are major expenses related to the disability, such as a change of residence, special foods, or assistive devices, caregivers can apply for a childcare allowance for added costs. A total of 10 contact days per child per year can be granted to caregivers of children who are <16 years of age and covered by the LSS. Contact days are for caregivers to learn how to support their child. In the event of severe mobility difficulties, a car allowance might be granted to help offset costs to acquire a modified vehicle. Respite care and relay services, where someone else temporarily assumes care of the child, can be granted. Home allowance can be applied for; however, parental income limits apply. If the child is 16–21 years of age and covered by the LSS, the caregivers can receive temporary parental benefits.

There is a transition period for adults moving from disability allowance to additional cost compensation. These will run in parallel until 2021, when disability allowance will be phased out. Adults >65 years with major chronic disabilities who need help with the activities of daily living can apply for attendance allowance to hire personal assistants. Car allowance might be granted to individuals >65 years who need a vehicle for transportation. Individuals 19–29 years of age who are unable to work full time for at least one year can apply for a time-limited activity and sickness compensation, which can be approved part or full time. Disability compensation can be granted to those aged 30–64 years whose working capacity has been permanently reduced. Individuals aged >65 years who receive activity or disability compensation and have a low income might be eligible for housing allowance, administered by the Social Insurance Agency. The amount granted depends on housing expenses, income and savings. Accommodation with special services includes group housing (full-time services), service residence (apartments with shared services) and adapted personal accommodation. Those participating in vocational rehabilitation can receive a rehabilitation cash benefit if unable to work. A benefit covering certain additional expenses related to receiving rehabilitation, such as travel, can also be granted. Rehabilitation cash benefit can be paid for part of, or the entire, day.

### Iceland

Caregivers can apply for tax-free care allowance benefits and, in some cases, for parental payments. The purpose is to compensate for disability-related expenses, such as treatment or training. Caregivers receiving these types of benefits can apply for reduction of Icelandic car taxes. Care allowance benefits are applicable up to 18 years of age. Parental payments are intended to compensate for loss of income for caregivers who are unable to partake in work or education because of their child’s disability and apply up to 18 years of age. Caregivers of children or adult children with disabilities may be entitled to refunds of travel expenses when services are not available locally. Generally, this applies to travel expenses for two trips annually but it can cover more frequent traveling. During hospitalizations (if the child resides at least 20 km from the hospital), caregivers can apply for partial refunds of accommodation cost. Supportive family is an option, which means the child temporarily visits another family. Personal support and assistance, not to be confused with UPA, is available to reduce social isolation and might include assistance to enjoy cultural or social activities. These services are provided by the municipalities. It is possible to apply for disability parking and for car allowance to buy or modify a car. The allowance may be granted every fifth year.

Most Icelandic adults with long-term support needs live in community homes. Adults (18–67 years) with disabilities can apply for disability pension administered by the Social Insurance Administration (SIA). Disability pension is composed of various benefit payments, depending on social or financial circumstances. The main disability benefit payments are disability pension, age-related disability supplement and guaranteed income. Amounts are income based. There are also household supplements, child allowance and compensation pensions. Individuals with disabilities have recently become legally entitled to UPA. UPA is based on person-to-person services, where the users themselves employ and arrange how services are rendered. Municipalities and UPA centres are responsible for granting personal assistance. UPA centres are cooperative communities where people with disabilities live semi-independently. Implementing UPA centres has been a complicated process, partly because of hesitation on the part of the municipalities. UPA is still being developed and has yet to become a widely used resource.

### Finland

The government, the municipalities and the federation of municipalities share the responsibility of providing services or benefits for people with disabilities. The municipalities assume the main responsibility for disability services. An ISP is created in collaboration with the municipality social services and the user. In this plan, all personal needs of the children and their families are documented and include modifications to the home, personal assistance, transportation, home care and other home services. Special needs due to IDs are also specified. Caregivers must apply separately for each service in the plan.

The Social Insurance Institution (KELA) provides financial support. Decisions of eligibility for disability allowances are based on the extent or type of care and assistance needed. There are two main allowances: disability allowance for individuals >16–64 years of age and disability allowance for children ⩽16 years. The allowance is to support everyday life, education and work and is divided into categories, depending on how much assistance is needed. The allowance can be granted for specific time periods, or with no set time limit, and is not contingent on income. Adolescents (aged 16–19 years), can apply for a Rehabilitation Allowance for Young Persons. The allowance is granted to those whose abilities to study or work are reduced, or if support is needed to study or participate in employment-based rehabilitation. If adolescents receive full-time disability pensions and need assistance in daily life, they can apply for care allowance for retirees. Other financial support includes reimbursements for medical expenses, travel costs and leisure-time assistance. A special care allowance compensates for the caregiver’s loss of earnings when they care for their child. Caregivers participating in courses or taking part in family rehabilitation are compensated for short-term loss of earnings.

Finland’s goal is that after 2020, no individuals with disabilities will live in institutions, but in their own homes. The municipalities provide grants for reasonable costs of home modifications and to purchase equipment and install it. Expenses related to modifications to improve accessibility can be covered. Personal assistance is provided for individual daily activities at home or in kindergarten or school and to assist with practical tasks for working adults. It is possible to have a support contact for leisure time, hobbies, community involvement and social contact. A ‘service voucher’ can be used to cover salaries for temporary assistants. KELA provides interpreting services for hearing, hearing-visual or communication difficulties. Compensation related to costs for extra clothing or special diets are available. If public transportation cannot be used, transport services can be rendered, which includes school transportation and to participate in activities. The right to free transport is based on distance, duration of the activity or difficulty in using public transportation. Municipalities can cover up to half of the purchase cost if a car is needed to transport children with severe disabilities. Disability parking permits can be granted. The pension system safeguards old age, incapacity to work and the death of a caretaker. Statutory pension consists of an occupational pension and a national pension. Those participating in vocational rehabilitation can apply for a rehabilitation allowance from KELA.

### Denmark

The welfare model is based on the principle that all citizens should be guaranteed certain fundamental rights if they encounter social problems. The public sector is responsible for the provision of social security benefits, social benefits and services. Most benefits and services are delivered by public-sector employees. The municipalities are responsible for planning and providing social services. The expenditure on financial assistance and rehabilitation is shared between the government and local authorities. If a child with CP needs special support, municipality officials will make an overall assessment (e.g. development, family relationships, school conditions, health conditions) to decide if help is needed. If it is, an ISP must be created. Within three months of a municipality becoming notified that a child has been diagnosed with CP, it must ensure the caregivers receive counselling and guidance. This is to strengthen the child and family’s development and wellbeing and to inform the family of public assistance available. Not all municipalities offer free family counselling. Before turning 18 years old, individuals with CP and their families must be offered advice on the availability of benefits to cover extra expenses, lost earnings and which benefits will end at the age of 18 years.

A person whose work ability is permanently and substantially impaired, and can only work a few hours per week, can be offered a flexible job scheme, where the municipality supplements the difference in salary. It is time limited for five years for those <40 years old. After 4.5 years, the municipality conducts an assessment and the person can remain at the same flexible job if they are still eligible. Those >40 years can get permanent flexible jobs if they meet the requirements. When it is evident that an individual’s work ability cannot be improved, the municipality can process a claim for early retirement. If approved, the individual can receive early retirement pension from age 40 years until retirement.

Caregivers can apply to the municipality to train their child in full or in part at home. Once the municipality has approved home training, caregivers who train their children in their own home can receive financial support for lost earnings. Home training is an alternative to a municipal offer and the municipality should assess the need of special support. Caregivers can receive financial support for additional costs incurred for special food, medications, clothing, leisure activities and disability-oriented courses for families. If there is a need for a car, caregivers can apply for an interest-free loan with 50% to be paid over eight years. Caregivers with a higher income will be required to repay more than 50%. A person with a disability or family members can apply for housing support. More support is available for those who require assistance 24 hours a day. Caregivers who need to can place their child in foster families, places of residence and day-care facilities without losing them.

Employers can be reimbursed for the cost of sickness benefit from the first day of illness because employees with disabilities at risk of being ill more often. There is also service support, which is offered by the municipality through job centres. The job centre can provide functional assistants to assist (e.g. lifting, driving, proofreading) the person in performing their jobs and provide assistive technology, if needed. Other service support includes leisure attendance, rehabilitation, mentorship, free physical therapy and counselling. Persons who have limitations in their ability to work and who do not receive other benefits under the Act on Active Employment Efforts can receive work-oriented rehabilitation. For public transport, special minibuses designed for wheelchair users are available, as is disability parking. Public transportation is discounted.

### Norway

The government and the municipalities share the responsibility of providing services and benefits for people with disabilities, including those with CP. Disability benefit is provided to ensure income when working capacity is reduced due to disability, resulting in a decrease in income by 50% and may be combined with part-time work. To be eligible for disability benefits, an assessment of health and functional level, a medical declaration and a meeting with an administrator in the Norwegian Labour and Welfare Administration (NAV) have to be conducted. The upper limit for disability benefit is 66% of the salary in the last five years and it may be permanent. An alternative is wage subsidies provided by the NAV, to stay employed and avoid disability benefit despite reduced working capacity. Wage subsidies may be time limited or permanent and serve to compensate the employer for hiring a person with reduced work capacity. The same process as for disability benefit proceeds wage subsidies and it has to be renewed every third month. The upper limit is 75% of full salary the first year and 67% thereafter.

Sick pay (maximum 52 weeks) is compensation to the employer when an employee is absent from work and a substitute is needed. Work assessment allowance may be provided after one year of sick leave, or at 19 years if working capacity is reduced with more than 50%, to ensure income. This time is generally limited to three years. The upper limit is 66% of the last year’s income. Basic benefit is meant to compensate for additional costs due to long-term disability. A medical report and documentation of extra costs are required. Caregiver benefit is a financial compensation for loss of income when caring for a relative in need of full-time care due to disability. Attendance allowance compensates for lost income when caring for a child who needs continuous care (including training allowance and care benefit days). Training allowance is to compensate for reduced income when attending courses or training necessary for taking care of the child. Housing grants cover additional housing expenses, such as improving accessibility. Special parking permits, car subsidies and customized transport are available for transport to work or school and for leisure travel (subsidized). Respite care is provided in private homes, in the home of the user or in sheltered housing. Personal assistants work for an individual user, where the user or family serve as the manager. Children can have assistants in kindergarten or school, and adults can have functional assistants at work, for example, to assist with proofreading. A support contact helps users have meaningful leisure time by accompanying them to leisure-time activities. A companion service can be provided for persons who need company when participating in cultural events. Technical assistive devices are available for free from the NAV. Activity devices are provided for individuals up to 25 years of age. Those 26 years or older receive limited annual grants for activity devices.

## Applying for social benefits

### Sweden

Persons with CP must apply for LSS themselves. If younger than 15 years, or if unable to apply, someone else who is legally allowed to may apply. Municipalities generally have assigned administrators who specialize in LSS. The LSS is a law of rights, so is possible to appeal a decision and a court can overturn decisions on LSS. To apply for personal assistance, a physician, with the assistance of other healthcare professionals, will need to fill out a form describing the person’s disability and the effect the disability has (i.e. medical certificate). This form is submitted with the application. It usually takes five months before a decision is finalized. Separate applications need to be submitted for the different social benefits. For some of these applications, additional certificates from physicians are required. Most of the social benefits are time limited and have to be reapplied for. This is the case even for individuals with lifelong disabilities that cannot be cured and tend to be stable.

### Iceland

Disability benefits and social support are provided by the government and the municipalities. However, it is generally delegated to different offices such as the SIA. It can therefore be complicated to navigate the system and apply for the support. The application for social benefits and support is sent to the SIA or to the individual’s municipality, as applicable. The application process for benefits requires a medical certificate each time and many of the benefits are time limited and have to be reapplied for. The individuals themselves, or someone legally entitled to do so on their behalf, fills out applications and questionnaires regarding their mental and physical health and functional ability. This has raised concerns because some find this degrading.

### Finland

Depending on the form of support, the application is sent to the municipality’s social services or the KELA. All benefits must be applied for by the individual, or by someone legally entitled to apply on their behalf. For some of the applications (transportation services, personal assistance, disability allowance), a medical certificate is required. The time to process an application is generally 4–7 weeks, and at most three months. For children, most of the benefits are time limited and need to be re-applied for; for adults, the benefits are generally valid until further notice. If applicants are not satisfied with the decision, it can be appealed, which is usually a long process.

### Denmark

The Central Disability Council advises the government on disability issues, whereas the municipal disability council advises the municipal council as needed. Individuals can contact case managers working on disability-related matters in the municipalities. The municipality must provide free counselling on what types of social benefits are available. If it lacks the expertise, the municipality can contact the National Knowledge and Special Advisory Organization, which provides specialized advice concerning disabilities. The municipalities make decisions about social benefits based on specific individual assessments (including medical certificates) of the recipient’s needs and circumstances. In practice, services and decisions vary between municipalities. Documentation is required as proof of disability and a case officer decides what documentation is needed. The support offered by the municipality depends on disability condition, age, personal expenses and justification to use assistive equipment. An applicant must show how the aid can compensate for impaired functioning. If an applicant disagrees with a decision, an appeal can be filed within four weeks.

### Norway

Most of the social benefits granted to persons with CP require applications to be submitted to the NAV and the person has to complete the application forms and renewals. Home-based services, housing allowance, respite care and UPA are applied for at the municipality. Disability benefits may be permanent, but most of the other support arrangements need renewals with defined intervals. The renewal applications require medical certificates or documentation of expenses. The processing time varies between regions and the type of benefit or support arrangement from 1–8 months. Decisions can be appealed.

## Discussion

Although there are national differences in terms of monetary amounts and terminology used, the similarities across the five countries regarding laws, social benefits offered for individuals with CP and the application processes are clear. All have ratified the CRPD and as such have indicated that they, at least in principle, acknowledge the human rights and the importance of the quality of life of individuals with disabilities. All have also ratified the OHCHR. All five nations aim to include the users’ needs and standpoints from the perspective of the ICF by recognizing that participation is important and that the physical or social environments are central to enabling participation. This is vital from a public health standpoint because limited accessibility and non-inclusion prevent individuals from partaking and benefitting from public health efforts. A shift from a more traditional, paternalistic system, to a system based on the ideology of independent living, where users are more in control over services, seems to be a trend across the Nordic countries, albeit at different levels and at different paces [26].

The principles of New Public Management have influenced the policies and the services available; privatization, freedom of choice for the users and the conceptualization of service users as consumers are common [26]. In the Nordic countries, the governments and municipalities share the responsibility of providing services and social benefits to those with CP who are eligible. When more responsibility falls on the municipalities, it also creates inequalities, because the priorities may differ between wealthy and less wealthy municipalities. Reforms related to social benefits for people with disabilities seem frequent across the five countries, likely due to high costs of services [26] and because of complaints of user organizations on how claims of social benefits are evaluated.

The application process for benefits requires a medical certificate. Individuals or caregivers also need to fill out applications with detailed and quite intimate descriptions of daily functioning. In all Nordic countries, many of the social benefits are time limited and need to be reapplied for after certain periods of time. This is understandable, given that certain disabilities fluctuate over time, and because the type and frequency of services needed might be age dependent. In the context of CP, which is a lifelong disability that is managed, not cured, the rationale for re-evaluations could benefit from discussion and reconsideration. The underlying damage that causes CP is non-progressive [12] and although comorbidities change over time, individuals with CP generally remain in the same GMFCS, MACS and CFCS categories. It is highly unlikely that they will need fewer services over time [32–35]. The cost-effectiveness of having individuals re-apply, busy, short-staffed, high-paid physicians write even more medical certificates, and government or municipality officials re-evaluate the same individuals warrant discussion and ideally scientific inquiry. Some of the countries have a shortage of neuro-paediatricians, the medical subspecialty generally responsible for writing the medical certificates for children and it needs to be considered if their time would be better spent treating patients. All the Nordic countries have a severe lack of rehabilitation specialists for adults with CP. In addition to being time consuming and costly, anecdotally, it is stressful for the individuals with CP and their families. It is considered meaningless to document, every time, that the child still has CP and it affects daily life. To be reminded, over and over, that the child might not improve is emotionally stressful. Clearly, checks and balances need to be in place to avoid that social benefit systems are taken advantage of. However, the re-evaluations of people with CP (and similar disabilities) do not seem to serve as a check of misuse of the system. It might rather reflect misunderstanding or lack of knowledge on the part of the governments and municipalities, or perhaps a hesitation to treat individuals with different medical diagnoses differently. This may lead them to primarily being treated as disabled, instead of the persons they are, with individual goals and needs. Nevertheless, all Nordic countries have laws pointing out the importance to base decisions on individual needs.

Laws are one thing, availability and reality another. Families with children with disabilities in the Nordic countries should have ISPs to guide coordinated and needs-based services. Research shows that these plans exist, but are not necessarily used [36–38]. According to the National Association of IDs in Iceland (personal communication Bryndís Snæbjörnsdóttir, 18 March 2020), members find the evaluation of impairment to be inadequate, resulting in fewer, or insufficient, benefits. Even though individuals have the right to support, barriers to service provision exist and result in service delays, something that is not limited to Iceland. Whereas the definition of a ‘perfect system’ to assess and distribute social benefits for individuals with CP may lie in the eye of the beholder, and frankly is impossible to achieve, it is still relevant to ponder how equitable and fair the application process is. Separate applications need to be submitted for the different benefits and it seems legitimate to wonder if, and how, parental socioeconomic status, grit, health and determination play a part in who is able to fill out and file applications, as well as the physician’s experience and skills in writing medical certificates [39]. Immigrant families have a double challenge when navigating the Nordic healthcare system [40]. A Norwegian study shows that even if the families are grateful and appreciate the systematic follow-up of their children, they found it was exhausting to navigate the processes to gain access to the services to which they are entitled. Experience and knowledge of the official making decisions on social benefits likely matter as well. Nevertheless, the resources allocated to people with disabilities are far greater than for many other countries that are not welfare states [41].

## Abbreviations

Abbreviations used in the article are presented in Table II.

**Table II.** List of abbreviations.

Abbreviation	Explanation	Brief description
CFCS	Communication Function Classification System	A five-level classification system describing everyday communication for children with cerebral palsy. A child at level I communicates easily, at level V the child is seldom able to communicate effectively.
CP	Cerebral palsy	A group of permanent disorders in the development of movement and posture attributed to non-progressive disturbances in the developing foetal or infant brain. The severity of motor disability varies widely, and cerebral palsy often occurs with secondary conditions.
CRPD	Convention on the Rights of Persons with Disabilities	A United Nation human rights treaty aiming to protect and promote human rights for people with disability. It stresses the right of persons with disabilities to be involved in preparing and making decisions on issues that concern them. Nations that ratify this convention are bound to it by international law.
GMFCS	Gross Motor Function Classification System	A five-level classification system describing the self-initiated movements of children with cerebral palsy. Level I indicates a mild affection of gross motor function, level V the most severe mobility issues.
ICF	International Classification of Functioning, Disability and Health	The World Health Organization framework and model for describing and organizing functioning and disability.
ISP	Individual Service Plan	A living document that changes over time to reflect the shifting priorities of the family, the child’s developmental stage, transition planning and progress toward goals and objectives (with some variation depending on country).
KELA	The Finnish Social Insurance Institution	Responsible for basic security for all residents in Finland. The service covers areas of social security such as family benefits, housing benefits, financial support for students, health insurance, rehabilitation, disability benefits, basic unemployment security and basic pensions.
LSS	Act Concerning Support and Service for Persons with Certain Functional Impairments (Sweden)	The law that regulates support and service to persons with functional disabilities in Sweden. Strives to enable the same opportunities to participation in society for all.
MACS	Manual Ability Classification System	A five-level classification system describing how children with cerebral palsy use their hands in everyday activities. Level I indicates an easy handling of objects, at level V the child is unable to handle objects.
NAV	Norwegian Labour and Welfare Administration	Responsible for welfare benefits and social security for Norwegian residents. The service covers services such as disability pension, work assessment allowance, unemployment benefits, sickness benefits and retirement pension. There are several legislations and statutes covering the service activities.
OHCHR	Convention on the Rights of the Child	A United Nations’ treaty that sets out the human rights for children in four general principles: all children are equal, entitled to a good life, the views of the child shall be considered and the interest of the child are primary in decision-making. Nations that ratify the convention are bound to it by international law.
SIA	The Social Insurance Administration of Iceland	Responsible for social benefits in Iceland. Financed by the State Treasury. The service covers services such as pension insurance, health insurance and occupational injury insurance. Includes all residents in Iceland with certain restrictions, for example regarding age and disability.
UPA	User-controlled personal assistance	An arrangement based on legislation that in turn is based on the person’s right to independent living. Gives persons with longstanding and high need for assistance a flexible ability to independent living.
UN	United Nations	An intergovernmental organization that aims to maintain international peace and security, develop friendly relations among nations, achieve international cooperation and be a centre for harmonizing the actions of nations.
VISO	National Knowledge and Special Advisory Organization (Denmark)	Under the National Board of Social Services, its purpose is to assist municipalities, citizens, regional and private providers with counselling advice and investigation in the most specialized and complicated cases in the social and special education fields.
WHO	World Health Organization	A specialized agency of the United Nations responsible for international public health.
